# Postprandial Referred Shoulder Pain: A Case Report

**DOI:** 10.7759/cureus.25535

**Published:** 2022-05-31

**Authors:** Keaton Ott, Joe Iwanaga, Aaron S Dumont, Marios Loukas, R. Shane Tubbs

**Affiliations:** 1 Department of Structural and Cellular Biology, Tulane University School of Medicine, New Orleans, USA; 2 Department of Neurosurgery, Tulane University School of Medicine, New Orleans, USA; 3 Anatomical Sciences, St. George's University, St. George, GRD; 4 Department of Neurosurgery, Neurosurgery and Ochsner Neuroscience Institute, Ochsner Health System, New Orleans, USA

**Keywords:** eating, shoulder, pain, anatomy, diaphragm

## Abstract

The precise mechanism of referred pain is not well understood; however, diaphragmatic irritation is a well-known etiology of referred pain. Left side referred pain due to diaphragmatic irritation is most commonly attributed to splenic laceration i.e. Kerr's sign. Here, we report an unusual case of left-sided referred pain that followed eating. An adult male presented vague and chronic left shoulder pain that followed eating. The pain was described as a deep boring type of discomfort that was poorly localized to the region deep to the acromion and extended superomedially along the upper fibers of the trapezius muscle. The pain was present immediately after eating heavy meals and always abated approximately 30 minutes later. There was no history of previous surgery and physical examination was unremarkable. CT examination of the abdomen and thorax did not show any pathology or anatomical variations that would result in such referred pain. Although the exact etiology of this case is unclear, the most likely cause would be left-sided diaphragmatic irritation from the stomach after eating. The current literature does not enclose reports pertaining to similar findings. Although unusual and seemingly rare, postprandial referred shoulder pain should be considered by clinicians alongside other causes of referred shoulder pain when presented with shoulder pain without an obvious musculoskeletal or neural etiology.

## Introduction

Referred pain can be defined as pain from a noxious stimulus originating in one area of the body which is perceived in another area of the body [1]. The prototypical example of referred pain is angina pectoris due to coronary artery disease, wherein ischemia of cardiac muscle is perceived as pain commonly in the left side of the chest, medial aspect of the left arm, and left neck or jaw. Another common site of referred pain with important diagnostic value for thoracic and abdominal disease are the shoulders [1]. Referred shoulder pain, attributed to irritation of phrenic nerve afferent fibers innervating the diaphragm and its associated serous membranes, can have numerous etiologies ranging from hemoperitoneum to pericarditis to foreign bodies. We present an unusual case of chronic referred pain to the left shoulder region reported only after eating.

## Case presentation

A 52-year-old healthy male presented for routine medical examination. The chief complaint was referred pain over the left shoulder region only after eating. The pain was described as being vague and chronic (greater than 20 years) in nature, and always being a deep boring type of discomfort that was poorly localized to the region deep to the acromion which extended superomedially along the upper fibers of the trapezius muscle. However, the pain could never be alleviated with shoulder position or palpation/massage of these regions. Moreover, analgesics such as ibuprofen or acetaminophen never altered the severity or duration of the pain. Specifically, the pain was present immediately after eating heavy meals and always abated approximately 30 minutes later. Neither the type of food nor the time of eating had any influence on the referred pain. Additionally, the position of the patient never altered the duration or severity of the referred pain. 

The patient’s past medical history included hypercholesterolemia and familial hypertension both of which were treated adequately with medications. There was no history of previous surgery. On physical examination, the patient’s motor and sensory exams were within normal limits. The neurological examination was non-focal. On thoracic examination, there were no murmurs, wheezing, or signs of airway disease. The head and neck examination was found to be within normal limits. Lastly, the abdominal examination was found to be normal with no bruits, tenderness, or masses identified. 

**Figure 1:**
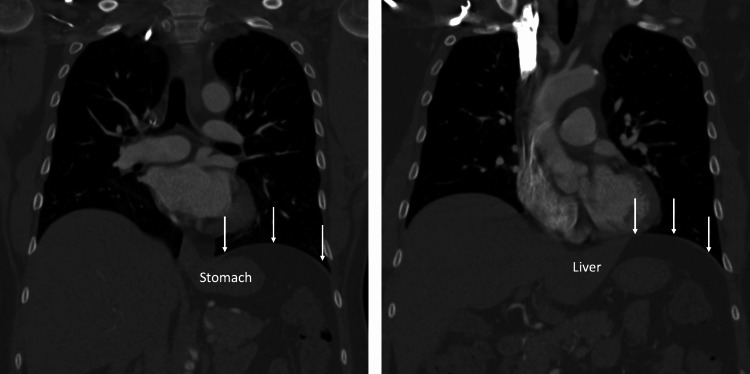
CT examples of the diaphragmatic region in the coronal plane noting the absence of pathology in this region. Arrows: diaphragm

**Figure 2:**
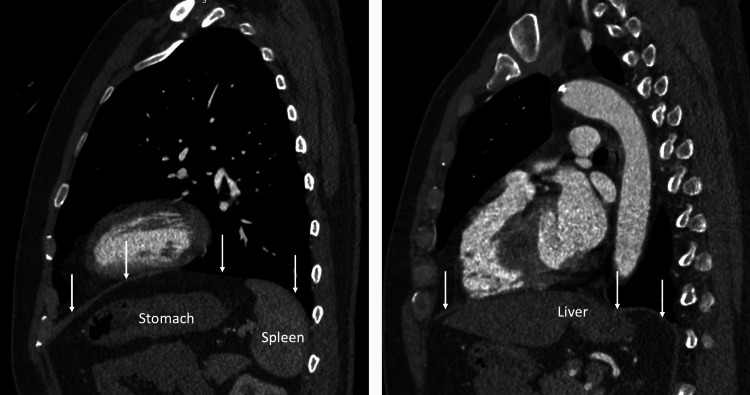
CT examples of the diaphragmatic region in the sagittal plane noting the absence of pathology in this region. Arrows: diaphragm

A CT was performed to evaluate the region around the diaphragm including the abdomen. There was no intrathoracic or intra-abdominal pathology (e.g., hiatal hernia) or anatomical variations noted. The stomach was in a normal position and no masses or abnormal fluid collections were found in the abdomen or, more specifically, near the diaphragm (Figure 1, 2).

## Discussion

We report a case of referred left shoulder pain in a patient without pathology or anatomical variations of the left side of the diaphragm. To our knowledge, this is the first reported case of referred pain to the left shoulder after eating. We propose that such a case represents transient diaphragmatic irritation/displacement by an overdistended stomach. 

The precise mechanism of referred pain is not well understood; however, a prevailing theory proposes that afferent fibers from different areas, visceral or somatic, converge onto common neuronal cell bodies in the spinal cord, resulting in central misinterpretation of the location of the noxious stimulus and perception of pain in an unstimulated area [1]. This may explain why referred pain commonly presents in specific segmental dermatomal or myotomal patterns. Using referred shoulder pain as an example, phrenic afferent fibers innervating the diaphragm and associated serous membranes terminate in the dorsal horns of C3-5 spinal cord levels alongside afferent fibers from the supraclavicular nerves (C3-4) and certain C5 ventral ramus branches such as the axillary or suprascapular nerves, which together innervate the skin, muscle, and joints of the shoulder [2]. Irritation in the diaphragmatic region eliciting referred pain in the shoulder thus reflects this common spinal cord level termination of their sensory fibers. In our case, the vague involvement of the upper trapezius might also point to its dual innervation via C3-C4 fibers from the cervical plexus that have, historically, been thought to convey proprioceptive sensation from this muscle.

Considering the varied structures surrounding the diaphragm and with phrenic nerve afferents innervating the diaphragm as well as portions of the parietal pleura, fibrous and serous pericardium, and parietal peritoneum, it should come as no surprise that a myriad of conditions may be the cause of referred shoulder pain [2]. Above the diaphragm, cases of referred shoulder pain due to pericarditis [3], lower lobe pneumonia [4], and Pancoast tumor [5] among others have been reported. Below the diaphragm, cases of referred shoulder pain due to gastric perforation [6], pancreatitis [7], and liver abscess [8] among others have been reported. The particularly asymmetrical anatomy of the abdomen lends itself to often predictable lateralization of referred shoulder pain, as is the case with left shoulder pain due to irritation of the left hemidiaphragmatic and related parietal peritoneum and hemidiaphragm from hemoperitoneum secondary to splenic rupture, a well-recognized physical finding referred to as Kehr’s sign [9]. Iatrogenic etiologies have also have been reported in cases presenting with referred shoulder pain. Dialysate inflow has been found to elicit shoulder pain in some peritoneal dialysis patients, worsened in the supine position or with deep inspiration, presumably due to subdiaphragmatic irritation of the peritoneum [10]. Similarly, shoulder pain resulting from peritoneal tubing lodged in a subdiaphragmatic position has been described as a complication of ventriculoperitoneal shunt placement [11]. 

## Conclusions

The present case report highlights the possibility of referred shoulder pain postprandially due to gastric distension. We are unaware of similar reports of such a phenomenon in the literature. Although unusual and seemingly rare, postprandial referred shoulder pain should be considered by clinicians alongside other causes of referred shoulder pain when presented with shoulder pain without an obvious musculoskeletal or neural etiology. 
